# Elucidating the anti-hypertensive mechanisms of Uncaria rhynchophylla-Alisma plantago-aquatica L: an integrated network pharmacology, cluster analysis, and molecular docking approach

**DOI:** 10.3389/fchem.2024.1356458

**Published:** 2024-02-29

**Authors:** Tong Yin, Han Zhang, Xingfang Liu, Dongfeng Wei, Cong Ren, Liangyu Cui, Yukun Li, Linshuang Wang, Jiaheng Wang, Zhiwei Zhao, Dasheng Liu, Liying Wang, Xuejie Han

**Affiliations:** ^1^ Institute of Basic Research in Clinical Medicine, China Academy of Chinese Medical Sciences, Beijing, China; ^2^ Guang’anmen Hospital, China Academy of Chinese Medical Sciences, Beijing, China; ^3^ Research Department, Swiss University of Traditional Chinese Medicine, Bad Zurzach, Switzerland

**Keywords:** hypertension, traditional Chinese medicine, Uncaria rhynchophylla, Alisma plantago-aquatica L., network pharmacology, molecular docking, bioactive compounds, drug discovery

## Abstract

**Background:** With the increasing global prevalence of hypertension, a condition that can severely affect multiple organs, there is a growing need for effective treatment options. Uncaria rhynchophylla-Alisma plantago-aquatica L. (UR-AP) is a traditional drug pair used for treating hypertension based on the liver-kidney synergy concept. However, the detailed molecular mechanisms underlying its efficacy remain unclear.

**Methods:** This study utilized an integrative approach combining network pharmacology, cluster analysis, and molecular docking to uncover the bioactive components and targets of UR-AP in the treatment of hypertension. Initially, we extracted data from public databases to identify these components and targets. A Protein-Protein Interaction (PPI) network was constructed, followed by enrichment analysis to pinpoint the bioactive components, core targets, and pivotal pathways. Cluster analysis helped in identifying key sub-networks and hypothesizing primary targets. Furthermore, molecular docking was conducted to validate the interaction between the core targets and major bioactive components, thus confirming their potential efficacy in hypertension treatment.

**Results:** Network pharmacological analysis identified 58 bioactive compounds in UR-AP, notably quercetin, kaempferol, beta-sitosterol (from Uncaria rhynchophylla), and Alisol B, alisol B 23-acetate (from Alisma plantago-aquatica L.), as pivotal bioactives. We pinpointed 143 targets common to both UR-AP and hypertension, highlighting MAPK1, IL6, AKT1, VEGFA, EGFR, and TP53 as central targets involved in key pathways like diastolic and endothelial function, anti-atherosclerosis, AGE-RAGE signaling, and calcium signaling. Cluster analysis emphasized IL6, TNF, AKT1, and VEGFA’s roles in atherosclerosis and inflammation. Molecular docking confirmed strong interactions between these targets and UR-AP’s main bioactives, underscoring their therapeutic potential.

**Conclusion:** This research delineates UR-AP’s pharmacological profile in hypertension treatment, linking traditional medicine with modern pharmacology. It highlights key bioactive components and their interactions with principal targets, suggesting UR-AP’s potential as a novel therapeutic option for hypertension. The evidence from molecular docking studies supports these interactions, indicating the relevance of these components in affecting hypertension pathways. However, the study acknowledges its limitations, including the reliance on *in silico* analyses and the need for *in vivo* validation. These findings pave the way for future clinical research, aiming to integrate traditional medicine insights with contemporary scientific approaches for developing innovative hypertension therapies.

## 1 Introduction

Globally, hypertension ranks as the most prevalent and burdensome chronic non-communicable disease (Grassi et al., 2024). The NCD Risk Factor Collaborative Group, analyzing 1,201 studies with 104 million participants from 1990 to 2019, reported a significant increase in hypertension prevalence (Collaboration et al., 2021). Furthermore, hypertension is known to cause damage to various target organs, leading to conditions such as stroke, kidney disease, and ischemic heart disease (Zhou et al., 2021). Consequently, enhancing the efficacy of hypertension treatment and actively controlling its risk factors are imperative to mitigate organ damage and improve patient quality of life.

Traditional Chinese Medicine (TCM) offers several advantages, including low drug resistance, therapeutic stability, protection of target organs, symptom relief, improved long-term survival chances, and fewer adverse effects (Li, 2022). Given these benefits, East Asian countries, particularly China, are increasingly focusing on medications derived from traditional medicine. In this context, herbal prescriptions for treating hypertension warrant attention. Data mining-based studies indicated that liver-kidney co-therapy is a common strategy in managing hypertensive disorders (Jie et al., 2021; Wenlong, 2022). *Uncaria rhynchophylla* (UR), targeting the liver meridian, and *Alisma plantago-aquatica L.* (AP), targeting the kidney meridian, from the UR-AP pair, emblematic of this approach Moreover, UR and AP are frequently utilized in prescriptions by renowned veteran Chinese medicine practitioners, as evidenced in data mining studies (Xiaotian et al., 2021; Yichu, 2021; Shudong, 2022). However, the complex chemical composition and obscure molecular mechanism of UR-AP limit its clinical application, necessitating a deeper understanding of its mechanism in treating hypertension.

**FIGURE 1:**
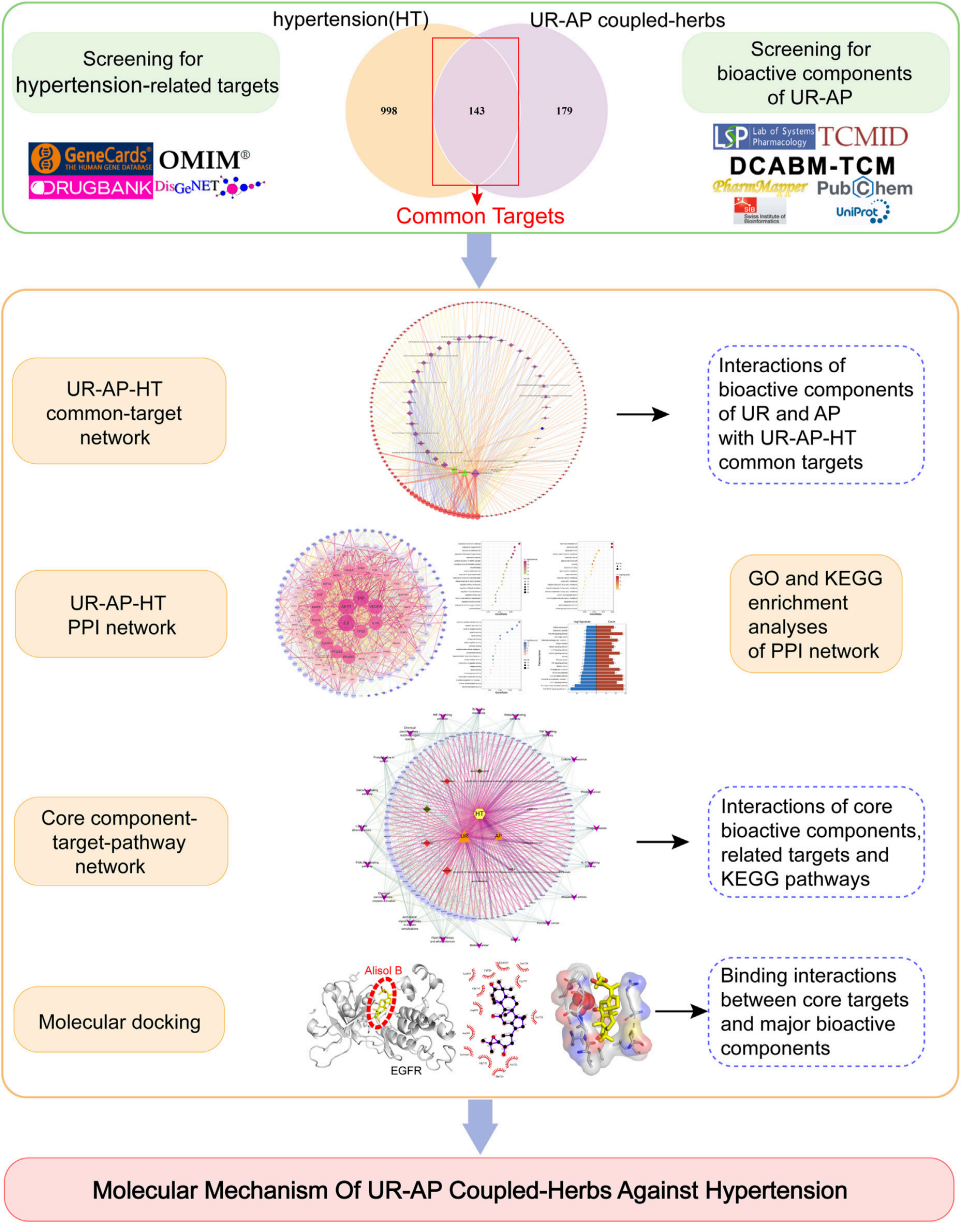
Flow chart of the whole study to reveal the mechanism of UR-AP against hypertension.

Network pharmacology, analyzing drug-target-disease interactions (Jiashuo et al., 2022), is particularly suitable for Chinese medicines characterized by component complexity and multiple interaction pathways. Therefore, network pharmacology cannot be ignored in the mechanism research of TCM. This approach aligns well with the multi-target network philosophy of TCM. Moreover, molecular docking, which simulates ligand-receptor binding to assess interaction stability and optimal binding modes, effectively complements network pharmacology. Therefore, this study aims to identify the bioactive components, core targets, and key signaling pathways of UR-AP in combating hypertension by constructing a Protein-Protein Interaction (PPI) network, performing cluster and enrichment analysis, and validating interactions through molecular docking (Figure 1).

## 2 Methods

### 2.1 Identification of bioactive components in UR-AP

Bioactive components of UR-AP were identified using the TCMSP (http://tcmspw.com/tcmsp.php) (Ru et al., 2014), TCMID (http://119.3.41.228:8000/tcmid/) (Huang et al., 2018), and DCABM-TCM (http://bionet.ncpsb.org.cn/dcabm-tcm/) (Liu et al., 2023). The ADME model, alongside published literature (Bai et al., 2021a; Bai et al., 2021b; Zhai et al., 2021; Liu et al., 2022), guided the setting of thresholds for oral bioavailability (OB) ≥ 30% and drug-likeness (DL) ≥ 0.18 to screen for bioactive components.

### 2.2 Identification of core targets of UR-AP

Core targets related to UR-AP’s bioactive ingredients were gathered from TCMSP, Swiss Target Prediction (http://www.swisstargetprediction.ch/) (Gfeller et al., 2013), and PharmMapper (http://www.lilab-ecust.cn/pharmmapper/) (Wang et al., 2017). Canonical SMILES format was obtained from PubChem (https://pubchem.ncbi.nlm.nih.gov/) (Kim et al., 2023). Gene mapping for the corresponding target was performed using Uniprot (https://www.uniprot.org/) (Coudert et al., 2023; UniProt et al., 2023), where gene name, IDs, and symbols were standardized and duplicates removed.

### 2.3 HT-related targets

Targets related to hypertension (HT) were collated from GeneCards (https://www.genecards.org/) (Stelzer et al., 2016), the DisGeNet database (https://www.disgenet.org/) (Pinero et al., 2020), Online Mendelian Inheritance in Man (OMIM, https://www.omim.org/) (Rasmussen et al., 2020), and Drug Bank (https://go.drugbank.com/) (Wishart et al., 2006). “Hypertension” and “essential hypertension” served as keywords. Data from different databases were merged after removing duplicates, and standardization of HT-related target names was conducted using UniProt.

### 2.4 UR-AP-HT common-target network

Integration of data and construction of the common-target network were accomplished using Cytoscape 3.7.1. The Network Analyzer plugin computed the degree values. A Venn diagram, created using R 4.2.1, facilitated the identification common targets of UR-AP and HT. After that, import the common targets into Cytoscape to build the UR-AP-HT common target network. Analysis of the main targets and bioactive components of UR-AP against HT according to the degree.

### 2.5 PPI network and evaluation

To illuminate the role of targets at the phylum level, the String database (https://cn.string-db.org/) (Szklarczyk et al., 2021) was used to produce the protein-protein interaction (PPI) network of UR-AP-HT common targets. Set the minimum confidence score to 0.400 to obtain a highly reliable PPI network relationship. Afterward, degree (DC), betweenness (BC), closeness (CC), eigenvector (EC), information (IC), local average connectivity-based method (LAC), network (NC), subgragh (SC) in CytoNCA were used to screen core targets.

### 2.6 Cluster analysis

MCODE is a plug-in for applying the K-means clustering algorithm. Further analysis of the PPI network by applying the MCODE plug-in could not only obtain the clusters composed of similar targets but also infer the main targets. The conditions were set to Degree Cutoff = 2, Node Score Cutoff = 0.2, K-core = 2, and Max. Depth = 100.

### 2.7 GO and KEGG pathway enrichment analyses

Gene Set Enrichment Analysis (GSEA) aggregated functionally similar gene individuals, allowing for filtering and screening of relevant functionality and pathway information. Gene ontology (GO) and kyoto encyclopedia of genes and genomes (KEGG) pathway enrichment analyses were conducted using the GO knowledgebase and KEGG database (Liu et al., 2017). The GO knowledgebase (http://geneontology.org/) (Gene et al., 2021) covers cellular components, molecular functions, and biological processes. The KEGG database (https://www.genome.jp/kegg/) (Kanehisa et al., 2023) integrates information from genomics, biochemistry, and systemic functional omics, commonly used in the KEGG pathway. The BiocManager package of R 4.2.1 manages several other packages, among which the clusterProfiler package is used to interpret the functional characteristics of genomics data and provide up-to-date gene annotations (Wu et al., 2021). The org.Hs.e.g.,.db package was used to provide human genome-wide annotations. The screening was based on *p* < 0.05.

### 2.8 Molecular docking

Molecular docking assessed the binding energy and optimal binding modes between ligand (bioactive components) and receptor (core targets). The core targets and main bioactive components of UR-AP against HT were respectively selected as receptors and ligands for molecular docking. The RCSB Protein Data Bank (RCSB PDB) (http://www.rcsb.org/) (Burley et al., 2021) was used to download the 3D crystal structures and save them in pdb format, including MAPK1 (PDB ID:8AOJ), IL6 (PDB ID:1ALU), AKT1 (PDB ID:1UNQ), VEGFA (PDB ID:4KZN), EGFR (PDB ID:8A27), TP53 (PDB ID:3D06). Download mol2 structure files of bioactive components from TCMSP and convert them into pdb format files using OpenBabel 3.1.1. Remove water and add all hydrogens by using PyMol 2.2.0. Afterward, the pdb format file was imported into AutoDock Tools 4.2 for further preprocessing of receptors and ligands and exported as the qdbqt format file. Molecular docking using AutoDock Vina 1.2.0. The docking parameters were set to modes = 20 and exhaustiveness = 10. Binding energy less than -5kj/mol can be considered feasible for docking results (Li et al., 2019). Visualization of docking results with 2D and 3D diagrams was achieved by using LigPlot+ 2.2.7 and PyMol 2.2.0.

## 3 Results

### 3.1 UR-AP component-target network

**FIGURE 2:**
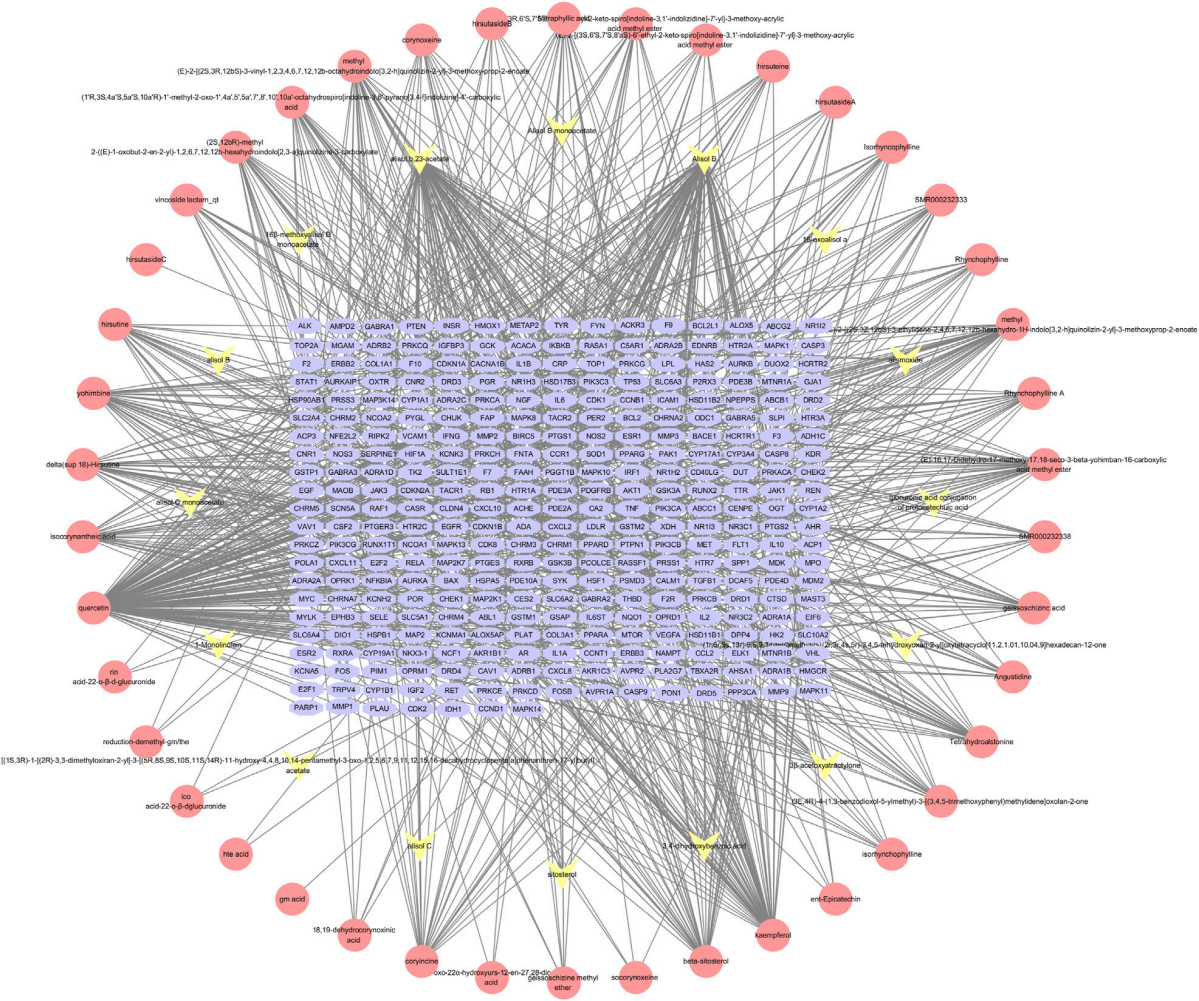
UR-AP component-target network: The pink nodes represent the bioactive components of UR, the yellow nodes represent the bioactive components of AP, and the blue nodes represent the core targets of UR-AP.

The components of UR-AP were obtained from TCMSP, TCMID, DCABM-TCM databases (Supplementary Table S1). After that, a total of 58 bioactive components were obtained after de-weighting and screening (Supplementary Table S2). These included 43 components from UR, 16 from AP, and 1 common component (sitosterol). Action targets for these 58 bioactive components were predicted using TCMSP, Swiss Target Prediction, and PharmMapper. In total, 322 targets of UR-AP (except cyanide) were predicted after removing duplicates (Supplementary Table S3). The UR-AP common-target network comprised 380 nodes and 1062 edges (Figure 2), with quercetin (degree = 151), alisol B (degree = 95), alisol,b,23-acetate (degree = 81), and kaempferol (degree = 62) identified as the primary bioactive components (Supplementary Table S4).

### 3.2 HT-related targets

To ensure the comprehensiveness of HT-related targets obtained, 1141 HT-related targets were acquired from 4 databases. Of these, 945 targets were obtained from GeneCards, 416 targets from DisGeNET, 25 targets from OMIM, and 42 targets from Drug Bank (Supplementary Table S5).

### 3.3 UR-AP-HT common-target network

**FIGURE 3:**
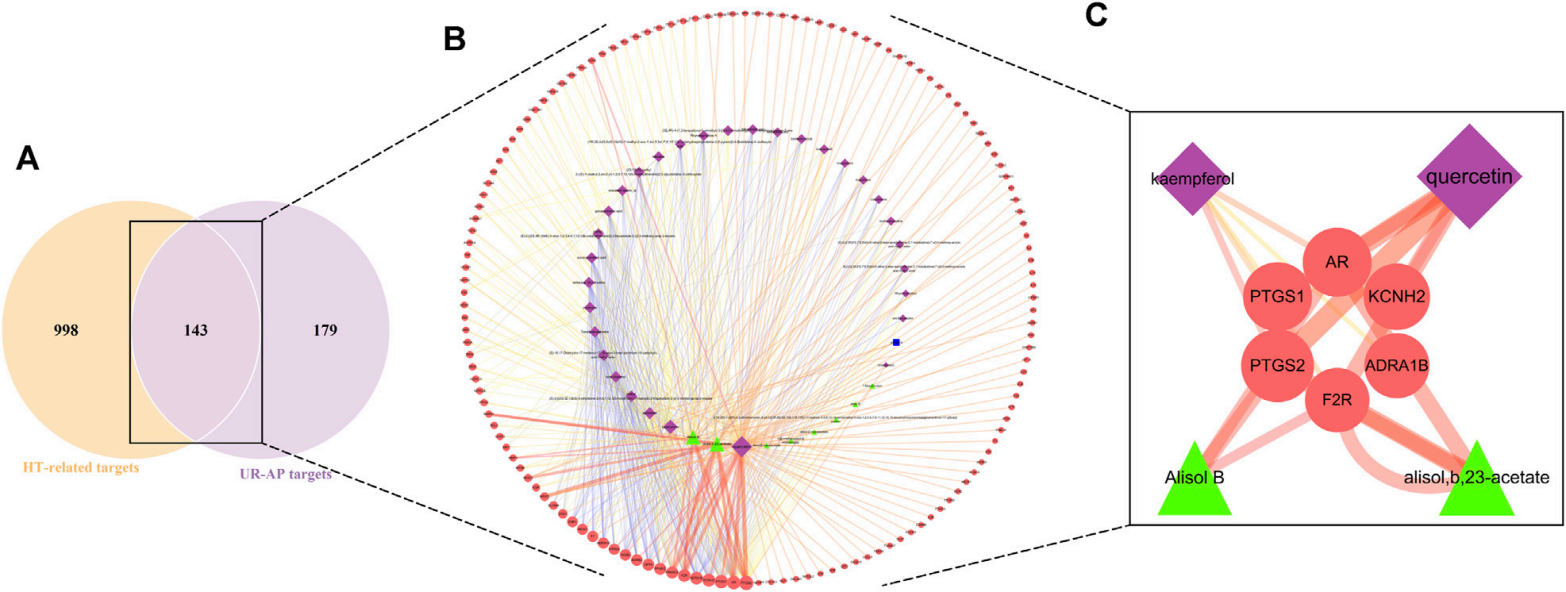
UR-AP-HT common-target network. **(A)** The intersection of the Venn diagram: 143 targets are common to HT and UR-AP. **(B)** UR-AP-HT common-target network. **(C)** Main bioactive components and potential targets. The purple nodes represent the bioactive components of UR, the green nodes represent the bioactive components of AP, and the blue nodes represent the bioactive components common to both UR and AP. The red nodes represent core targets. The node sizes were ordered by degree. The thickness and color shading of the edges were set according to the degree of interaction between the nodes.

The intersection of UR-AP core targets and HT-related targets identified 143 common targets (Figure 3A). The resulting network consisted of 183 nodes and 477 edges (Figure 3B), involving 32 bioactive components from UR, 9 from AP, and 1 common component. The main targets and bioactive components were analyzed according to degree values (Supplementary Table S6). The results indicated that there were four critical bioactive components for UR-AP treatment of HT, quercetin (degree = 84) and kaempferol (degree = 35) from UR, and alisol,b,23-acetate (degree = 38) and alisol B (degree = 36) from AP. In addition, PTGS2, AR, PTGS1, KCNH2, ADRA1B, and F2R were the main targets of UR-AP for HT treatment (Table 1) (Figure 3C).

**TABLE 1:** Core targets of UR-AP in treating HT.

NO	Gene name	Protein names	Degree
1	PTGS2	Prostaglandin G/H synthase 2	33
2	AR	Androgen receptor	27
3	PTGS1	Prostaglandin G/H synthase 1	27
4	KCNH2	Potassium voltage-gated channel subfamily H member 2	23
5	ADRA1B	Alpha-1B adrenergic receptor	21
6	F2R	Thrombin	20

### 3.4 UR-AP-HT PPI network

**FIGURE 4:**
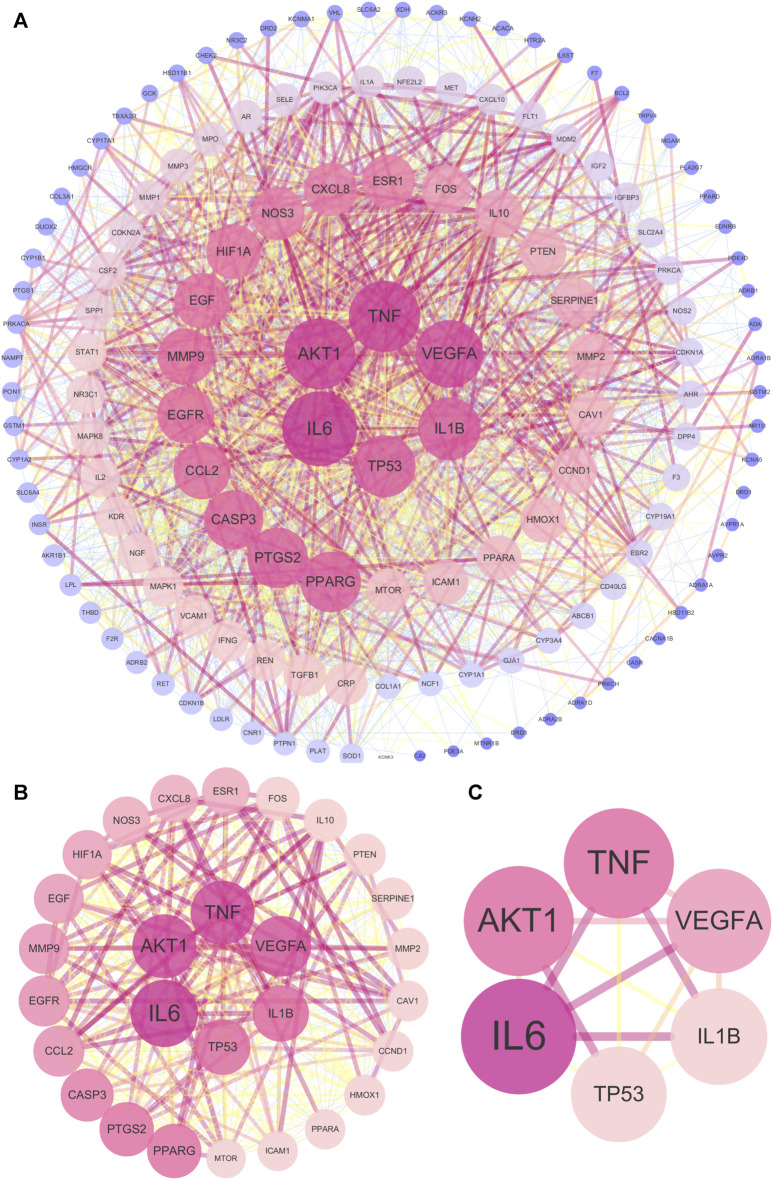
UR-AP-HT PPI network. **(A)** Total UR-AP-HT PPI network. **(B)** PPI network by the screening criteria of DC ≥ 55, BC ≥ 48.71780123, CC ≥ 0.534090909, EC ≥ 0.059021739, IC ≥ 10.76018715, LAC ≥18.775, NC ≥ 20.805268, SC ≥ 18778800000000000000. **(C)** PPI network by the screening criteria of DC ≥ 80, BC ≥ 366.8913084, CC ≥ 0.684466019, EC ≥ 0.159114137, IC ≥ 14.1830368, LAC ≥36.4516129, NC ≥ 69.88953295, SC ≥ 136440000000000000000. The size and color shading of the nodes were ordered by degree. The thickness and color shading of the edges were set according to the degree of interaction between the nodes.

The 143 common targets were imported into String database, and then 1 free target was discarded. Afterward, the PPI network included 142 nodes and 2296 edges (Figure 4A). The topological parameters of the UR-AP-HT PPI network were calculated, and the significant targets of the UR-AP-HT PPI network were obtained based on DC, BC, CC, EC, IC, LAC, NC, and SC (Supplementary Table S7) (Figures 4B, C). Finally, six important targets were obtained, namely, Interleukin-6 (IL6), Tumor necrosis factor (TNF), RAC-alpha serine/threonine-protein kinase (AKT1), Vascular endothelial growth factor A (VEGFA), Interleukin-1 beta (IL1B), Cellular tumor antigen p53 (TP53) (Figure 4C).

### 3.5 Cluster analysis

**FIGURE 5:**
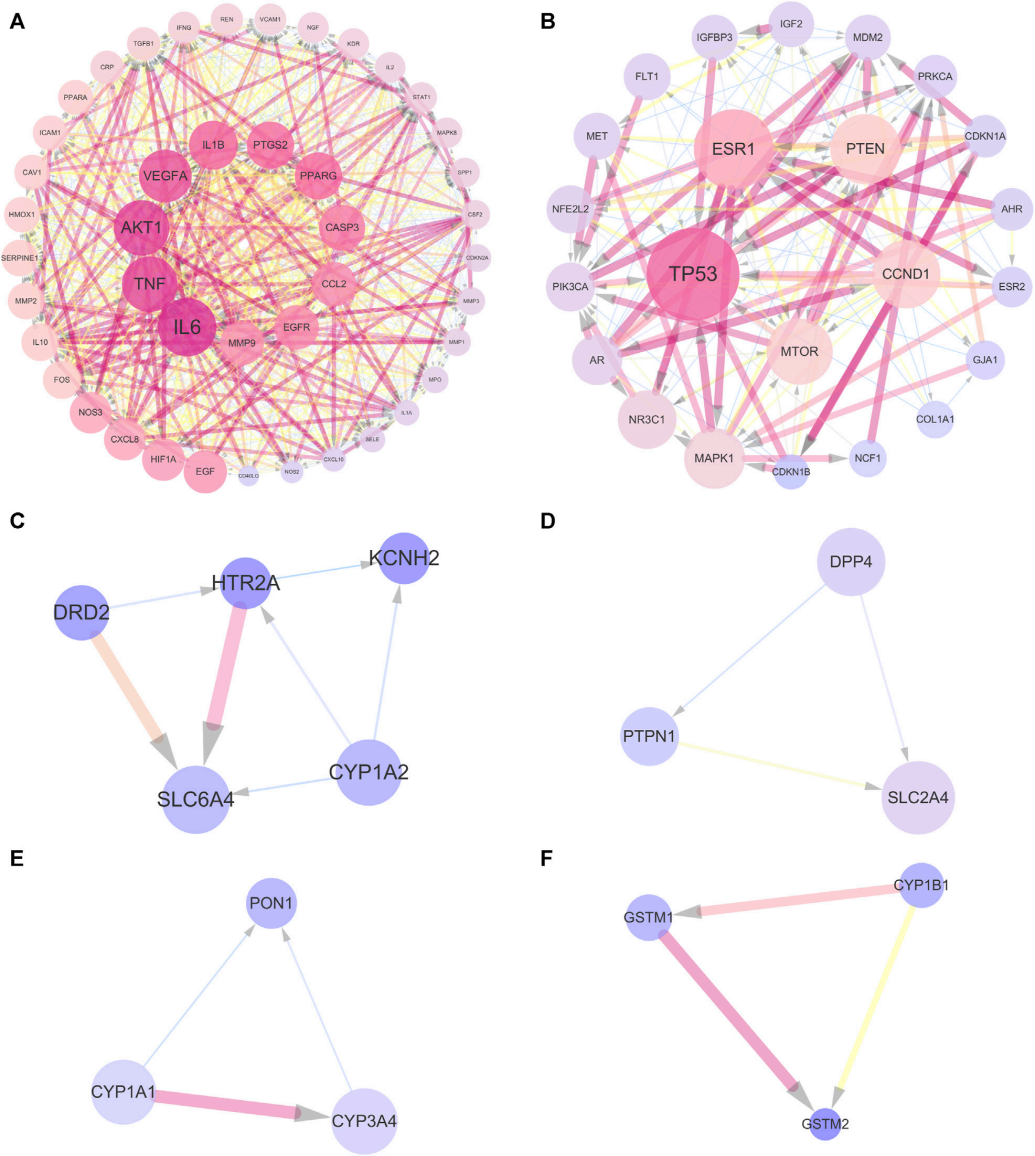
Cluster analysis of UR-AP-HT PPI network. **(A)** Cluster 1, composed of 44 nodes and 796 edges (score = 37.023). **(B)** Cluster 2, composed of 23 nodes and 139 edges (score = 12.636). **(C)** Cluster 3, composed of 5 nodes and 7 edges (score = 3.5). **(D)** Cluster 4, composed of 3 nodes and 3 edges (score = 3). **(E)** Cluster 5, composed of 3 nodes and 3 edges (score = 3). **(F)** Cluster 6, composed of 3 nodes and 3 edges (score = 3). The size and color shading of the nodes were ordered by degree. The thickness and color shading of the edges were set according to the degree of interaction between the nodes. The arrow pointed to the target.

In order to further analyze the 143 common targets of UR-AP and HT, find the key sub-networks, and speculate the main targets, the UR-AP-HT PPI network was further computationally analyzed by MCODE. The PPI network was divided into 6 clusters (Supplementary Table S8) (Figures 5A–F). The score of cluster 1 (score = 37.023) was far higher than the other clusters. The degree of the targets in cluster 1 was higher, such as IL6 (degree = 98), TNF (degree = 93), AKT1 (degree = 88), VEGFA (degree = 88), IL1B (degree = 81), PTGS2 (degree = 78), PPARG (degree = 78).

### 3.6 GO and KEGG pathway enrichment analyses of PPI network

**FIGURE 6:**
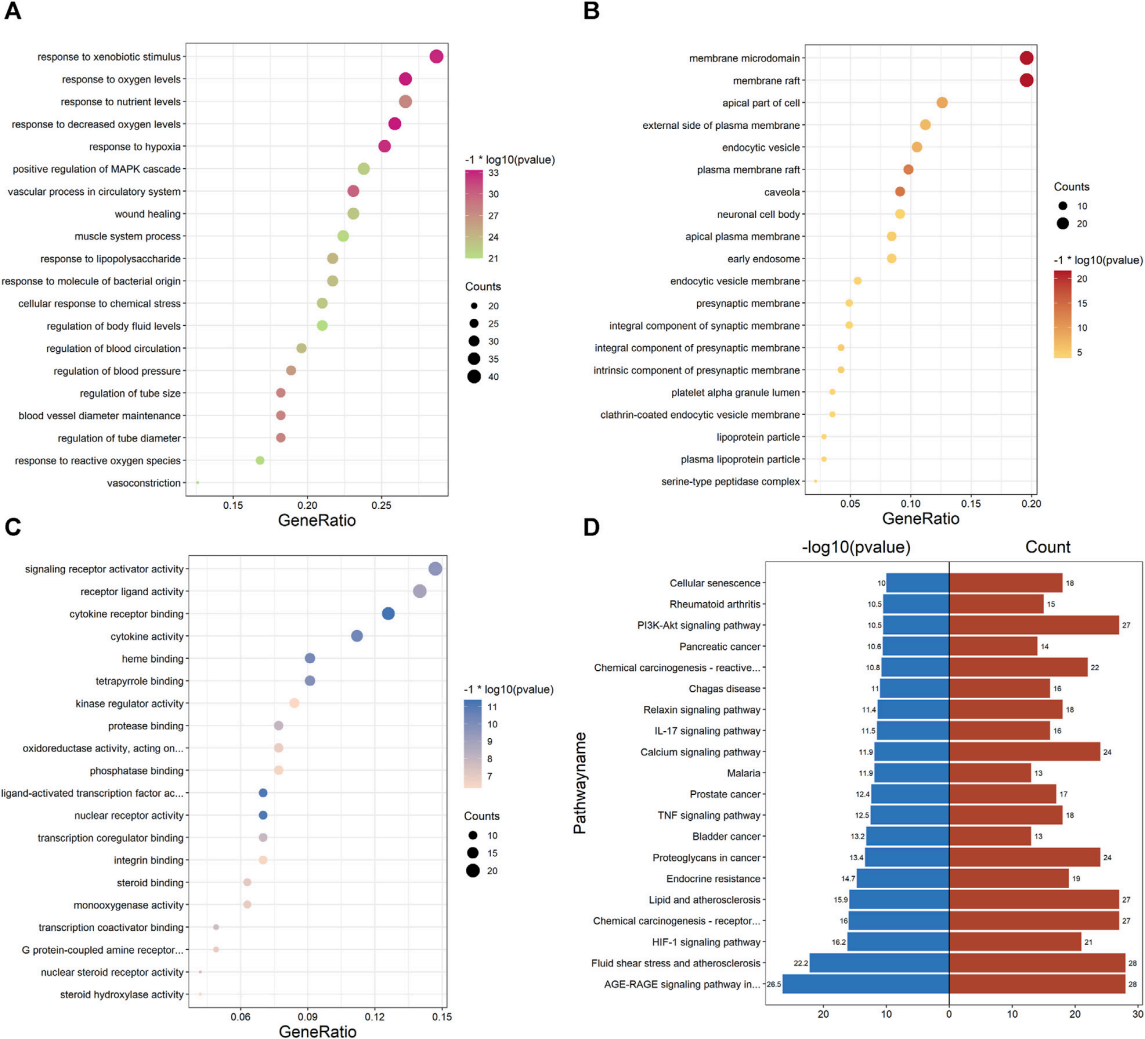
GO and KEGG pathway enrichment analyses of PPI network. **(A)** Bubble diagram of the top 20 biological processes. **(B)** Bubble diagram of the top 20 cellular components. **(C)** Bubble diagram of the top 20 molecular functions. The color was adjusted according to −1* log10 (*p*-value) and the size of the dots was adjusted according to the number of genes. **(D)** Bidirectional histogram of the top 20 KEGG pathways. Blue bars represent −1*log10 (*p*-value) and red bars represent the number of genes.

Enrichment analysis of the UR-AP-HT PPI network was obtained by applying the BiocManager package of R 4.2.1 (Supplementary Table S9). In GO enrichment analysis, 143 UR-AP-HT common targets were highly enriched in 2569 biological processes (BP), 82 cellular fractions (CC), and 206 molecular functions (MF) by setting *p*-values less than 0.05. The top 20 significant results for each were taken as shown in the figure (Figures 6A–C). Specifically, BP had a tight correlation with response to decreased oxygen levels, response to oxygen levels, and response to hypoxia, which indicates a possible role of UR-AP on HT in antihypoxia. Besides, there was also a strong correlation with vascular process in circulatory system, regulation of tube diameter, blood vessel diameter maintenance, regulation of tube size, response to nutrient levels, and regulation of blood pressure, suggesting that UR-AP may regulate blood pressure by modulating tubular diameter. CC is closely correlated with membrane raft and membrane microdomain, reflecting the possibility that UR-AP regulates blood pressure by acting on the cell membrane. MF was associated with cytokine receptor binding, cytokine activity, signaling receptor activator activity, and receptor ligand activity, suggesting that UR-AP may affect cytokine, signaling receptor, and receptor ligand activities. In addition, a total of 156 pathways were enriched by KEGG enrichment analysis, and the top 20 important pathways were shown in the figure (Figure 6D). The results indicated that UR-AP may regulate atherosclerosis through Fluid shear stress and atherosclerosis, Lipid and atherosclerosis, as well as through AGE-RAGE signaling pathway in diabetic complications and calcium signaling pathway to regulate endocrine (Table 2). UR-AP also regulates signaling pathways such as the HIF-1 signaling pathway and TNF signaling pathway.

**TABLE 2:** Partial results of KEGG pathway enrichment analysis.

ID	Description	pvalue	Count	geneID
hsa05418	Fluid shear stress and atherosclerosis	6.22E-23	28	BCL2/NOS3/AKT1/TNF/MAPK8/HMOX1/ICAM1/SELE/VCAM1/GSTM1/GSTM2/KDR/VEGFA/FOS/MMP2/MMP9/TP53/CAV1/IL1B/CCL2/PLAT/THBD/IFNG/IL1A/NCF1/NFE2L2/PIK3CA/TRPV4
hsa05417	Lipid and atherosclerosis	1.40E-16	27	BCL2/CASP3/PRKCA/PPARG/NOS3/AKT1/TNF/MAPK8/MMP1/CYP1A1/ICAM1/SELE/VCAM1/MMP3/FOS/MMP9/MAPK1/IL6/TP53/IL1B/CCL2/CXCL8/NCF1/NFE2L2/CD40LG/PIK3CA/LDLR
hsa04933	AGE-RAGE signaling pathway in diabetic complications	2.92E-27	28	BCL2/CASP3/PRKCA/TGFB1/NOS3/AKT1/TNF/MAPK8/STAT1/ICAM1/SELE/VCAM1/VEGFA/CCND1/MMP2/MAPK1/IL6/F3/IL1B/CCL2/CXCL8/THBD/SERPINE1/COL1A1/IL1A/COL3A1/PIK3CA/CDKN1B
hsa04066	HIF-1 signaling pathway	6.20E-17	21	BCL2/PRKCA/NOS2/NOS3/AKT1/HMOX1/INSR/EGFR/VEGFA/CDKN1A/MAPK1/EGF/IL6/HIF1A/SERPINE1/IFNG/MTOR/PIK3CA/CDKN1B/VHL/FLT1
hsa04020	Calcium signaling pathway	1.37E-12	24	PRKACA/DRD1/HTR2A/ADRA1A/ADRA1B/ADRB2/PRKCA/NOS2/F2R/NOS3/KDR/MET/ADRA1D/ADRB1/NGF/EGFR/VEGFA/EGF/AVPR1A/TBXA2R/RET/EDNRB/CACNA1B/FLT1

### 3.7 GO and KEGG pathway enrichment analyses of the cluster

**FIGURE 7:**
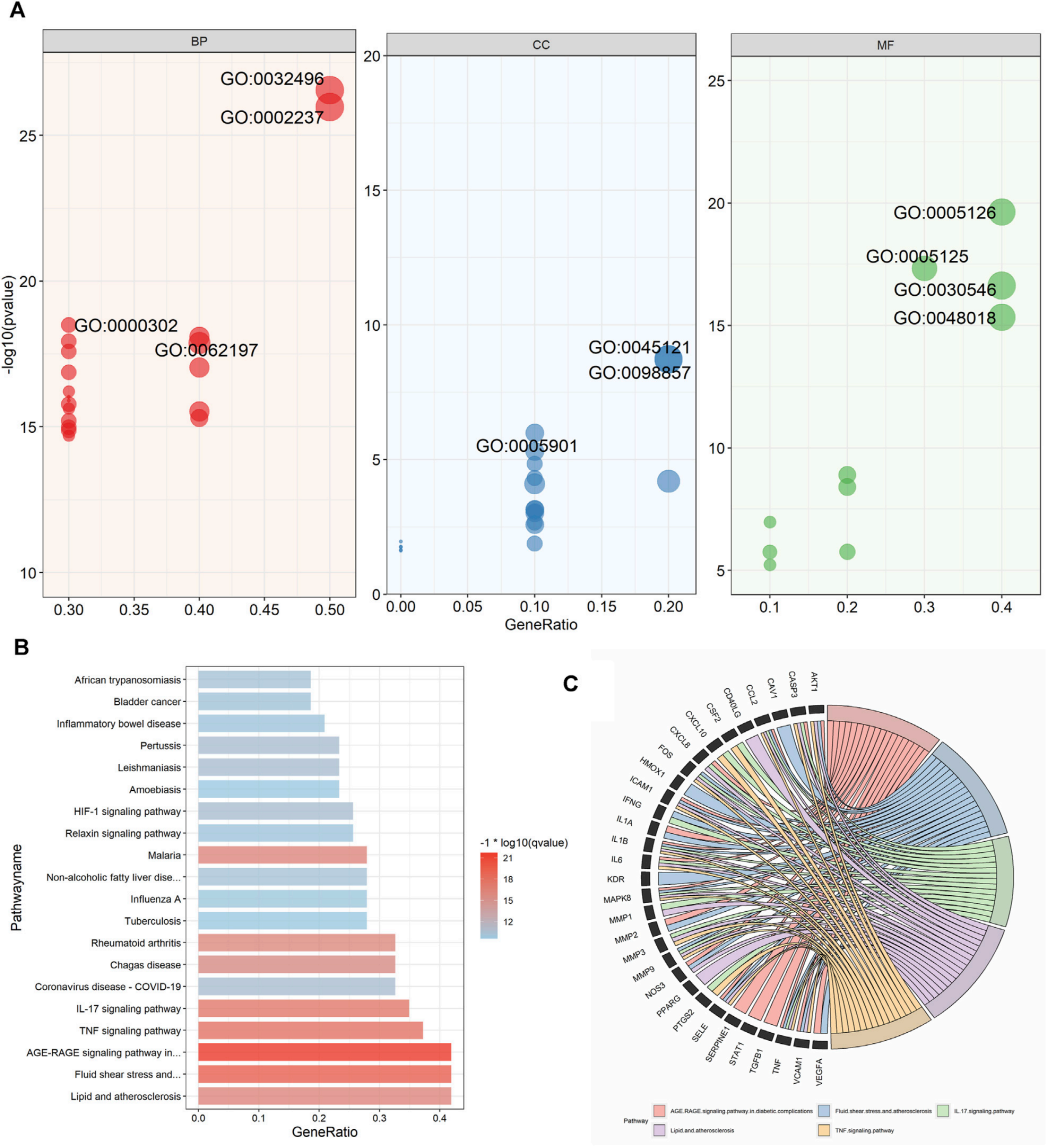
GO and KEGG pathway enrichment analyses of cluster 1. **(A)** The top 20 GO enrichment analysis results. The red ones represent the results of biological processes and show the ID of -log10 (*p*-value) > 18. The blue ones represent the results of cellular components and show the ID of -log10 (*p*-value) > 6. The green ones represent the results of molecular functions and show the ID of -log10 (*p*-value) > 15. The size of the points was adjusted according to the number of genes. **(B)** Histogram of the top 20 KEGG pathways. Color shading was adjusted according to −1*log10 (*p*-value). **(C)** String diagram of KEGG enrichment analysis results for cluster 1.

GO and KEGG enrichment analysis was performed on cluster 1 (score = 37.023), which had the highest score in the cluster analysis, to further obtain the functional information and pathway information of cluster 1 (Supplementary Table S10). In the GO enrichment analysis, 44 targets in cluster 1 were highly enriched in 2026 biological processes, 27 cellular components, and 115 molecular functions by setting *p*-values less than 0.05. The top 20 significant results for each were taken as shown in the figure (Figure 7A). Specifically, BP had a strong correlation with response to lipopolysaccharide, cellular response to lipopolysaccharide, which suggests a possible effect on lipopolysaccharide. As well as a close correlation with response to reactive oxygen species, response to oxidative stress, and response to decreased oxygen levels, suggesting a possible antioxidant effect of UR-AP against HT. CC is associated with membrane raft, membrane microdomain, etc., suggesting that it may act on the cell membrane to exert the effect. MF is tightly related to cytokine receptor binding, cytokine activity, signaling receptor activator activity, and receptor ligand activity, indicating that UR-AP may influence cytokines and signaling in the pathogenesis of HT. Furthermore, a total of 132 pathways were enriched by KEGG enrichment analysis, and the top 20 important pathways were shown in the figure (Figure 7B), and the top 5 important pathways and gene distribution were displayed (Figure 7C) (Table 3).

**TABLE 3:** The KEGG enrichment analysis of cluster 1.

ID	Description	pvalue	Count	geneID
hsa04933	AGE-RAGE signaling pathway in diabetic complications	3.47E-24	18	MAPK8/SERPINE1/IL6/TNF/IL1A/AKT1/VEGFA/VCAM1/SELE/CCL2/CXCL8/NOS3/IL1B/CASP3/STAT1/ICAM1/MMP2/TGFB1
hsa05418	Fluid shear stress and atherosclerosis	1.87E-21	18	MAPK8/TNF/IL1A/AKT1/VEGFA/VCAM1/SELE/CCL2/MMP9/FOS/IFNG/NOS3/IL1B/KDR/HMOX1/CAV1/ICAM1/MMP2
hsa04668	TNF signaling pathway	9.65E-20	16	MAPK8/IL6/TNF/AKT1/VCAM1/MMP3/SELE/CCL2/CXCL10/MMP9/FOS/IL1B/CSF2/CASP3/ICAM1/PTGS2
hsa04657	IL-17 signaling pathway	2.88E-19	15	MAPK8/IL6/TNF/MMP3/MMP1/CCL2/CXCL10/MMP9/FOS/CXCL8/IFNG/IL1B/CSF2/CASP3/PTGS2
hsa05417	Lipid and atherosclerosis	5.84E-18	18	MAPK8/IL6/TNF/AKT1/VCAM1/MMP3/PPARG/MMP1/SELE/CCL2/CD40LG/MMP9/FOS/CXCL8/NOS3/IL1B/CASP3/ICAM1

### 3.8 Core component-target-pathway network

**FIGURE 8:**
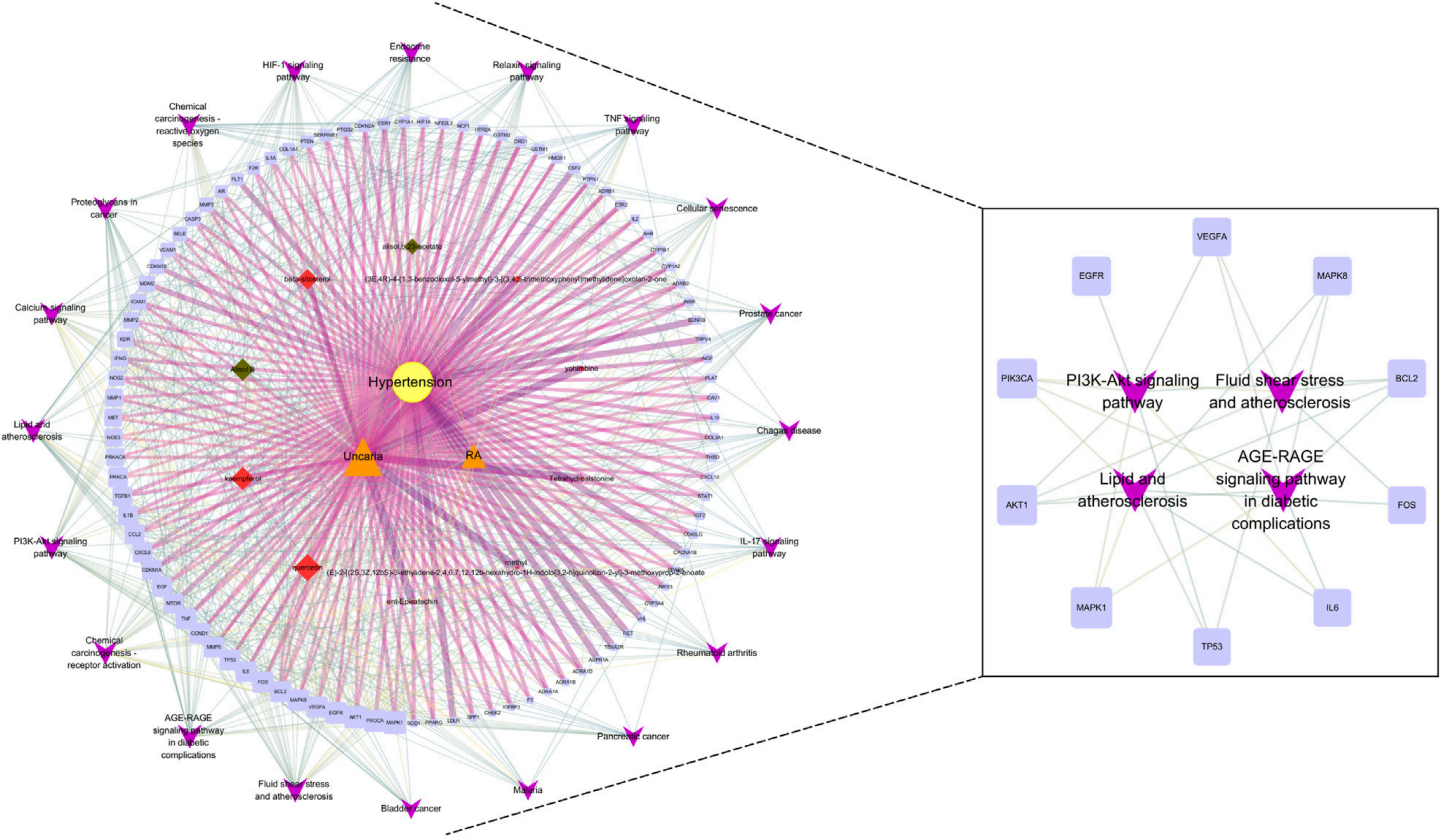
Core component-target-pathway network. The yellow nodes represent HT. The orange nodes represent UR and AP. The red nodes represent the bioactive components of UR, and the green nodes represent the bioactive components of AP. The blue nodes represent relevant targets. The purple nodes represent the top 20 signaling pathways from the enrichment analysis. The node sizes were ordered by degree. The thickness and color shading of the edges were set according to the degree of interaction between the nodes.

For further revealing the action mechanism of UR-AP for HT, 20 KEGG pathways, related targets, and bioactive components were constructed into a systematic and complex network, including 128 nodes and 731 edges (Figure 8). The core component-target-pathway network of UR-AP for HT was analyzed according to the degree values (Supplementary Table S11). The results indicated that there were mainly 3 key bioactive components in the core component-target-pathway network, including quercetin (degree = 40) and kaempferol (degree = 24) from UR, and Alisol B (degree = 21) from AP. Moreover, MAPK1, PIK3CA, AKT1, EGFR, VEGFA, MAPK8, BCL2, FOS, IL6, and TP53 were the top 10 significant targets.

**FIGURE 9:**
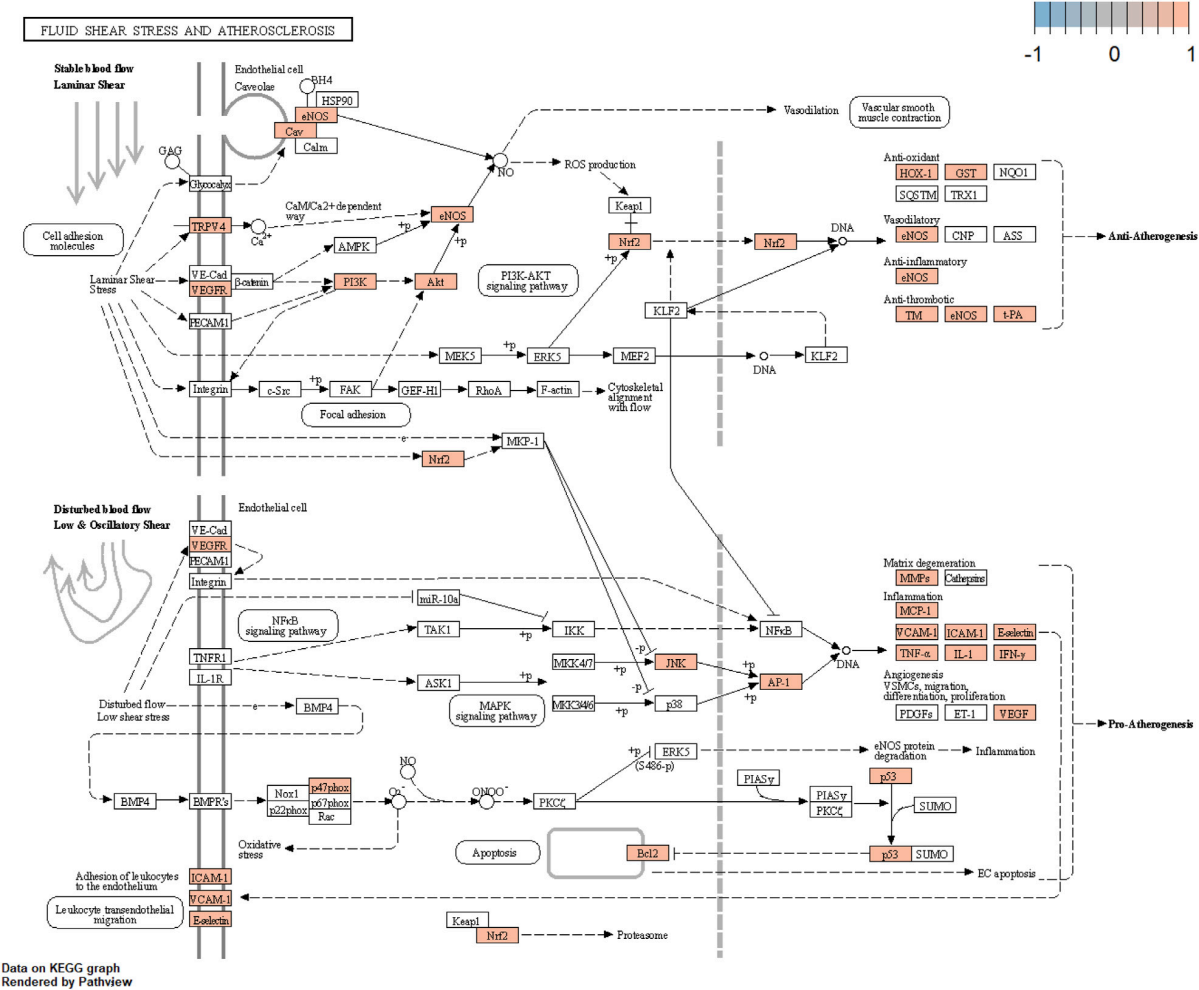
Fluid shear stress and atherosclerosis signaling pathway.

The core component-target-pathway network identified relevant signaling pathways including Fluid shear stress and atherosclerosis (degree = 28), AGE-RAGE signaling pathway in diabetic complications (degree = 28), PI3K-Akt signaling pathway (degree = 27), Lipid and atherosclerosis (degree = 27). One of the most significant pathways considered for UR-AP in treating hypertension according to the degree value is Fluid shear stress and atherosclerosis, indicating that UR-AP may exert hypotensive and antiatherogenic effects by acting on vascular endothelial cells to exert stable laminar shear stress, thus participating in vasodilation, antioxidant, anti-inflammatory and antithrombotic Sclerosis. The action path is shown in the (Figure 9).

### 3.9 Molecular docking

Combining the top 10 most important targets from the results of the core component-target-pathway network (MAPK1, PIK3CA, AKT1, EGFR, VEGFA, MAPK8, BCL2, FOS, IL6, TP53) with the top 10 most significant targets from the results of the PPI network and the cluster analysis (IL6, TNF, AKT1, VEGFA, IL1B, TP53, PTGS2, PPARG, CASP3, EGFR), we found that the duplicated targets were IL6, AKT1, VEGFA, EGFR, TP53. Due to the fact that MAPK1 was the most prominent target in the core component-target-pathway network, we concluded that MAPK1, IL6, AKT1, VEGFA, EGFR, and TP53 were the core targets of UR-AP against HT. In addition, there were 8 duplications between each of the top 10 most important core bioactive components of the core component-target-pathway network and UR-AP-HT common-target network. Therefore, we selected that these 8 duplicated bioactive components (quercetin, alisol,b,23-acetate, alisol B, kaempferol, yohimbine, beta-sitosterol, methyl (E)-2-[(2S,3Z,12bS)-3-ethylidene ethylidene-2,4,6,7,12,12b-hexahydro-1H-indolo [3,2-h]quinolizin-2-yl]-3-methoxyprop-2-enoate, Tetrahydroalstonine) as small molecules (ligands), and MAPK1, IL6, AKT1, VEGFA, EGFR, TP53 as proteins (receptors) to perform molecular docking, with the aim of validating the interactions between core targets and bioactive components. Finally, 48 groups of receptor-ligand molecular docking results were obtained (Supplementary Table S12).

**FIGURE 10:**
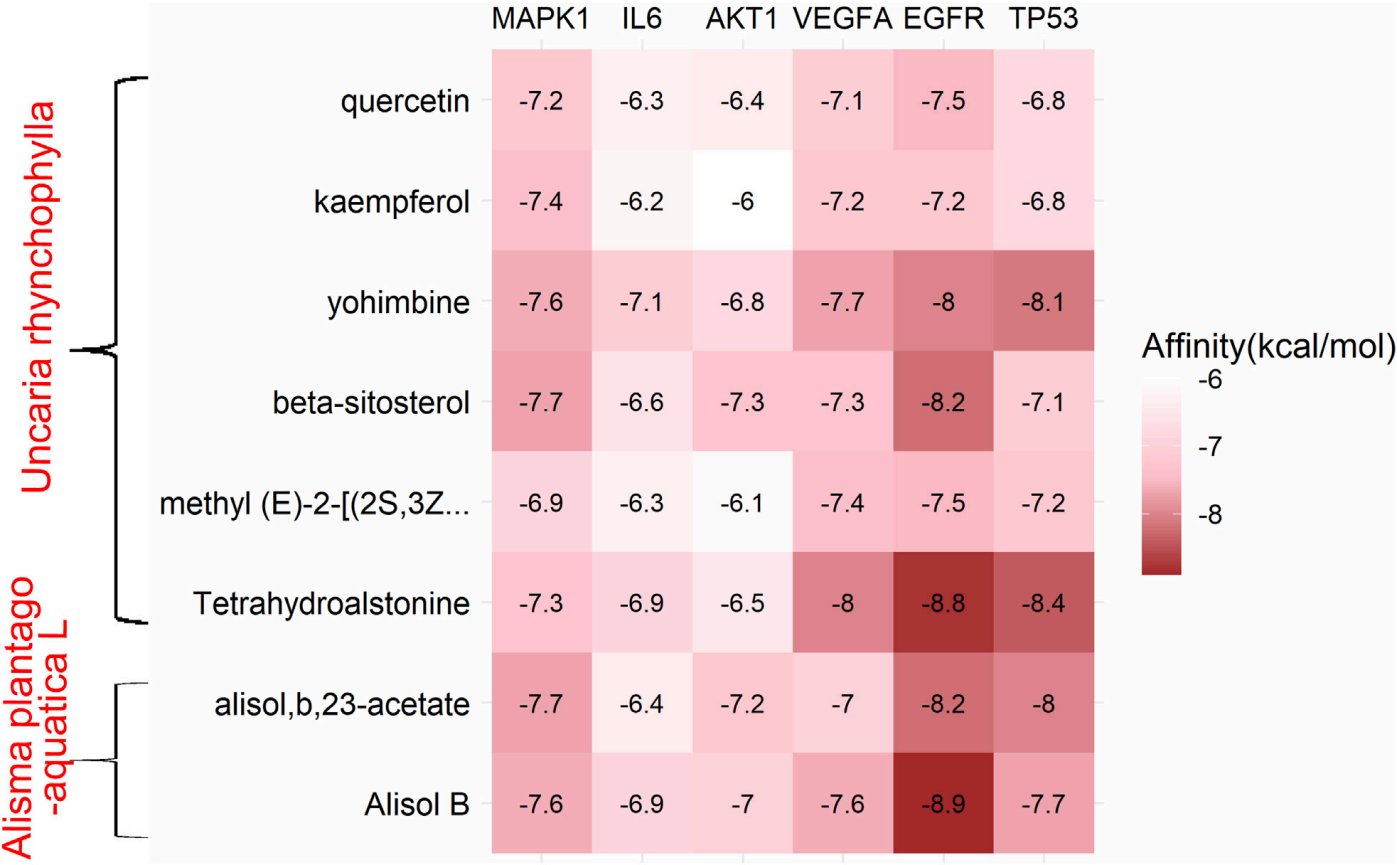
Heat map of the binding energy.

**FIGURE 11:**
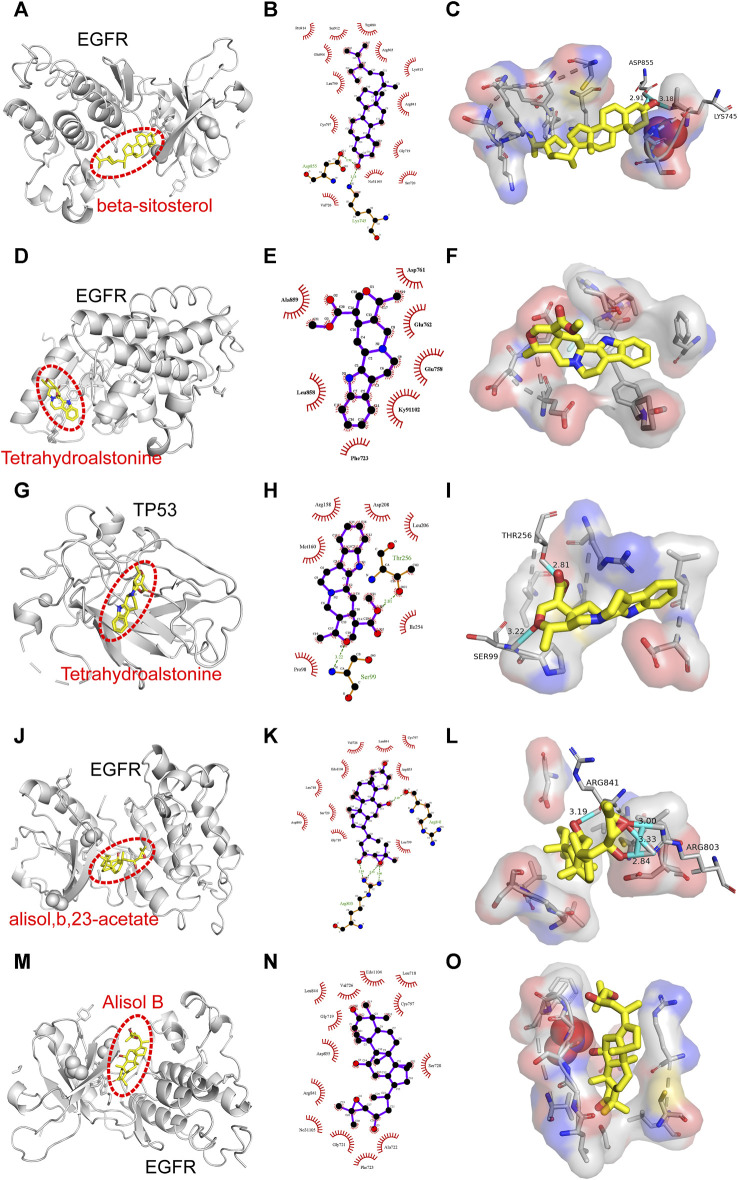
Molecular docking models of the lowest binding energy. **(A–C)** Molecular docking models of beta-sitosterol and EGFR. **(D–F)** Molecular docking models of Tetrahydroalstonine and EGFR. **(G–I)** Molecular docking models of Tetrahydroalstonine and TP53. **(J–L)** Molecular docking models of alisol,b,23-acetate, and EGFR. **(M–O)** Molecular docking models of Alisol B and EGFR. **(A, D, G, J, M)** Diagrams of the overall effect of molecular docking. **(A, D, G, J, M)** 2D diagrams of molecular docking. **(A, D, G, J, M)** 3D diagrams of molecular docking.

Binding energy less than −7.0 kcal/mol indicates superior binding activity, and the lower the binding energy, the better the docking effectiveness (Trott and Olson, 2010). Among the 48 groups of receptor-ligand docking results, there were 33 groups with the binding energy less than −7.0 kcal/mol, which indicated the strong binding interactions between the core targets and the bioactive components of UR-AP for HT treatment (Figure 10). The five groups with the lowest binding energy were selected to draw molecular docking models (Table 4) (Figure 11). The results indicated that beta-sitosterol was docked with 13 residues in EGFR (PRO914, SER912, TRP880, GLU906, ARG803, LEU799, LYS913, ARG841, CYS797, GLY719, NO31105, SER720, VAL726), and formed hydrogen bonds with ASP855 (2.91Å) and LYS745 (3.18Å) (Figures 11A–C). Tetrahydroalstonine was docked with seven residues forming hydrophobic interactions in EGFR (ASP761, ALA859, GLU762, GLU758, LEU858, KY91102, PHE723) (Figures 11D–F). Moreover, Tetrahydroalstonine was docked with six residues in TP53 (ARG158, ASP208, LEU206, MET160, ILE254, PRO98) and the hydrogen bonds were formed at PRO98 (3.22 Å) and THR256 (2.81 Å) (Figures 11G–I). Alisol,b,23-acetate was docked with 10 residues in EGFR (ASP800, GLY719, SER720, LEU718, EDO1104, VAL726, LEU844, CYS797, ASP855, LEU799), and formed hydrogen bonds with ARG803 (3.33Å, 3.00 Å, 2.84Å) and ARG841 (3.19Å) (Figures 11J–L). Alisol B was docked with 13 residues to form hydrophobic interactions in EGFR (PHE723, ALA722, GLY721, NO31105, ARG841, ASP855, SER720, GLY719, CYS797, LEU844, VAL726, EDO1104, LEU718) (Figures 11M–O). In addition, the docking energy of Alisol B with EGFR was the lowest (−8.9 kcal/mol) compared to other docking results, indicating that the Alisol B-EGFR complex is more stable than other targets.

**TABLE 4:** Molecular docking results in the lowest binding energy.

Ligand	Proteins	Residues	Hydrogen bonds	Affinity (kcal/mol)	RMSD	Dist from best mode	
						rmsd 1.b	rmsd u.b
beta-sitosterol	EGFR	PRO914,SER912,TRP880,GLU906,ARG803,LEU799,LYS913,ARG841,CYS797,GLY719,NO31105,SER720,VAL726	ASP855(2.91Å),LYS745(3.18Å)	−8.2	1.442	0.000	0.000
Tetrahydroalstonine	EGFR	ASP761,ALA859,GLU762,GLU758,LEU858,KY91102,PHE723	—	−8.8	0.001	0.000	0.000
Tetrahydroalstonine	TP53	ARG158,ASP208,LEU206,MET160,ILE254,PRO98	PRO98 (3.22Å),THR256 (2.81Å)	−8.4	0.000	0.000	0.000
alisol,b,23-acetate	EGFR	ASP800,GLY719,SER720,LEU718,EDO1104,VAL726,LEU844,CYS797,ASP855,LEU799	ARG803 (3.33Å,3.00Å,2.84Å),ARG841 (3.19Å)	−8.2	1.423	0.000	0.000
Alisol B	EGFR	PHE723,ALA722,GLY721,NO31105,ARG841,ASP855,SER720,GLY719,CYS797,LEU844,VAL726,EDO1104,LEU718	—	−8.9	1.124	0.000	0.000

## 4 Discussion

### 4.1 Hypertension: a global health challenge and the role of TCM

The rising incidence of hypertension, driven by factors such as dietary habits, lifestyle changes, and emotional stress, continues to be a global health concern (Rahimi et al., 2017). Symptoms like dizziness and headache significantly impact the quality of life of many patients. TCM, known for its efficacy in controlling blood pressure and alleviating symptoms, highlights the potential of developing new herbal treatments for HT. UR-AP, a common drug pair in TCM for HT treatment, is particularly notable. Although *Uncaria rhynchophylla* is widely recognized for its effectiveness in HT management, the complexities of the chemical composition of UR-AP and the specific components and targets involved in its action against hypertension are not fully understood (Dong et al., 2020; Tian et al., 2020; Deng et al., 2022). Therefore, this study is the first to use the network pharmacology approach to reveal the action mechanism of UR-AP in treating hypertension and to verify the interaction between core targets and bioactive components of UR-AP against hypertension with molecular docking.

### 4.2 Bioactive components and targets of UR-AP in hypertension management

In this study, 58 bioactive components and 322 targets of UR-AP were obtained by screening the online database. Additionally, 1,141 HT-related targets were gathered from disease target databases. The intersection of these targets using a Venn diagram led to the identification of 143 common targets, forming the basis for the UR-AP-HT common targets network. This network revealed that UR-AP’s therapeutic effects on hypertension are likely due to the synergistic action of various bioactive components. Based on the results of the UR-AP-HT common-target network and core component-target-pathway network, we first predicted that quercetin, kaempferol, beta-sitosterol from UR and Alisol B, alisol,b,23-acetate from AP were the main bioactive components of UR-AP against hypertension. The anti-hypertensive and cardioprotective effects of quercetin have been extensively studied. Quercetin can play a role in anti-hypertension by suppressing Ang II-stimulated VSMC proliferation (Wang et al., 2022). Quercetin may reduce oxidative stress, and minimize arterial endothelial cell dysfunction (Pereira et al., 2018). Quercetin was able to promote endothelial cell autophagy and reduce the blood pressure value of SHRs (Lin et al., 2020). Kaempferol (KFL) is a naturally occurring flavonoid with pleiotropic cardioprotective and antihypertensive effects. The pleiotropic cardioprotective effect of kaempferol can be attributed to the anti-inflammatory activity *in vitro* and animal studies (Dabeek and Marra, 2019; Zhao et al., 2023). Kaempferol can increase the phosphorylation of eNOS via the NO-cGMP-PKG pathway and induce endothelium-dependent vasorelaxation (Tettey et al., 2019). Beta-sitosterol has cholesterol-lowering and tissue-repairing effects and can be used to alleviate hyperlipidemia and atherosclerosis (Khan et al., 2022). Both alisol,b,23-acetate (AB23A) and alisol B, as alisol extracts, are effective ingredients in the treatment of atherosclerosis. AB23A and Alisol B may interfere with lipids and atherosclerosis by modulating PI3K-AKT and MAPK pathways, thereby treating Pregnancy-induced Hypertension and reducing liver and kidney injury (Liao et al., 2022). AB23A can regulate bile acid metabolism, lower cholesterol and alleviate atherosclerosis by increasing FXR-BSEP signaling (Fu et al., 2022).

To bridge the gap between these findings and clinical practice, it is essential to consider the feasibility and potential challenges of translating the identified gene targets into therapeutic interventions. The extensive research on the cardioprotective and antihypertensive effects of quercetin and kaempferol underscores their potential as foundational compounds in novel hypertension treatments. However, the clinical application of these bioactive components requires careful consideration of their bioavailability, pharmacokinetics, and safety profiles in human populations. Future studies should focus on the development of delivery mechanisms that enhance the efficacy of these compounds, rigorous clinical trials to establish therapeutic dosages, and comprehensive safety evaluations. This approach will ensure that the promising results of network pharmacology studies can be effectively translated into tangible benefits for patients with hypertension.

### 4.3 Mechanisms of action: core targets and pathways influenced by UR-AP

Synthesizing the results of the core component-target-pathway network, PPI network, and cluster analysis, we predicted that the core targets for UR-AP treatment of HT are MAPK1, IL6, AKT1, VEGFA, EGFR, and TP53. These have abundant interactions with other targets and are involved in additional signaling pathways. MAPK1 and AKT1 are able to facilitate vascular endothelial cell proliferation and angiogenesis, which is closely related to hypertension (Zhang et al., 2019). P53 is the protein product of TP53, which is implicated in atherosclerosis, VSMC growth, and cell death (Han et al., 2021). The p38 MAPK pathway is involved in blood pressure regulation and vascular injury (El-Sabbagh et al., 2023). The Akt pathway can be engaged in the differentiation of VSMC (Li et al., 2024). IL6 promotes smooth muscle cell proliferation and is implicated in hypertension through interactions with cytokines (Attiq et al., 2024). VEGFA is positively correlated with elevated blood pressure and is strongly associated with vascular damage and atherosclerosis (Braile et al., 2020). EGFR is related to cell proliferation, angiogenesis, and apoptosis inhibition (Luo et al., 2021).

### 4.4 Impact of UR-AP on vascular function and atherosclerosis

**FIGURE 12:**
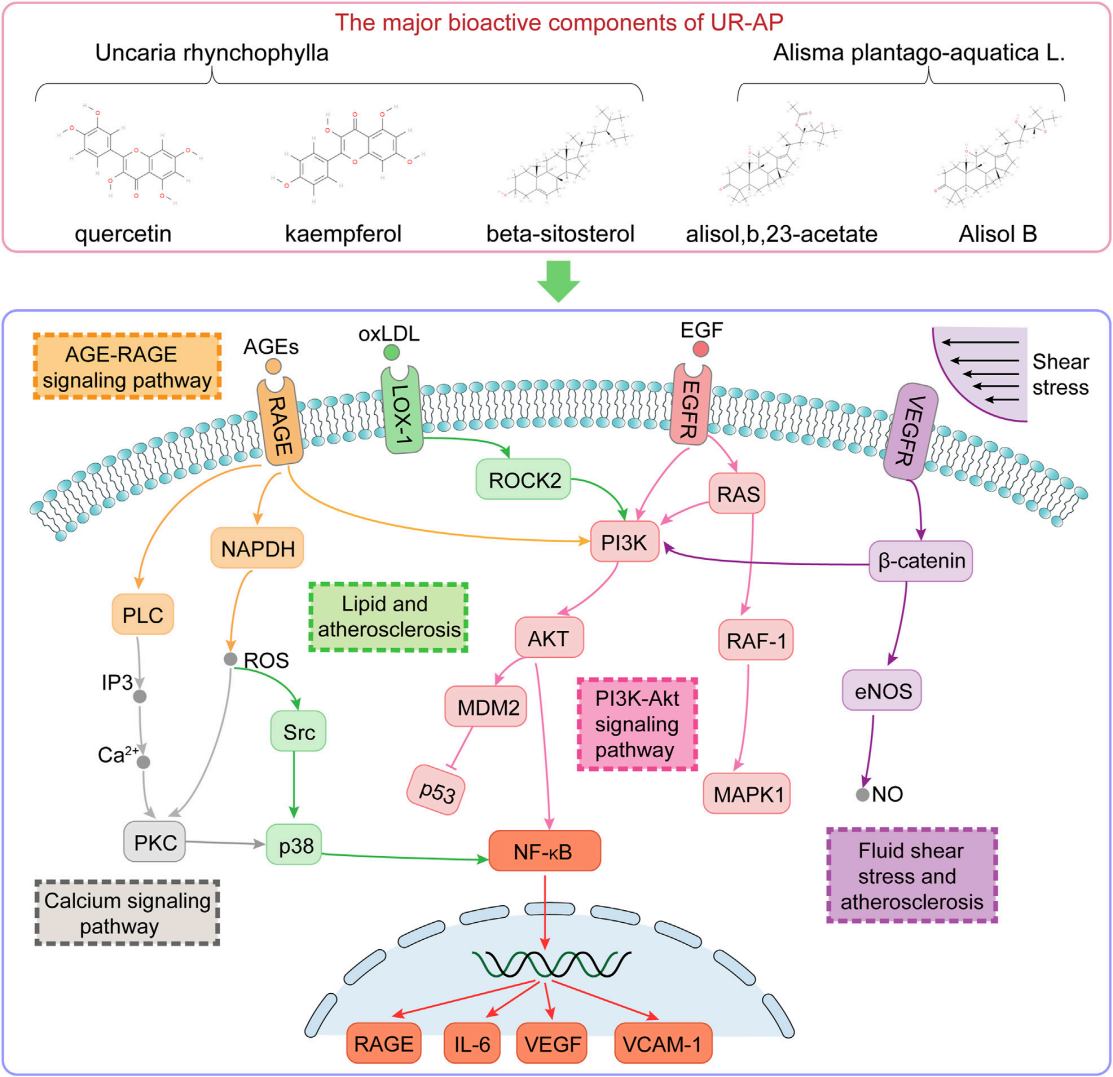
Illustration of the signaling pathways associated with the major bioactive components of UR-AP for the treatment of HT.

KEGG pathway enrichment analysis and core component-target-pathway network identified important signaling pathways involved in vasoconstriction, endothelial function, atherosclerosis, inflammatory response, and apoptosis (Figure 12). AGE-RAGE signaling pathway may mediate diabetic vascular complications (Burr et al., 2020). The binding of NAPDH, a product of RAGE, to AGEs enhances oxidative stress, stimulates the production of cytokines and growth factors, affects smooth muscle proliferation and apoptosis, causes atherosclerosis as well as vasoconstriction, and thus increases blood pressure (Eid et al., 2018). In addition, the combination of AGE and RAGE activates signaling pathways such as PI3K-Akt pathway and Calcium pathway, which can further activate NAPDH and lead to cell damage or even apoptosis (Sanajou et al., 2018). Calcium signaling pathway plays an important role in vasoconstriction and diastole. Elevated intracellular Ca^2+^ concentration induces vasoconstrictive effects (Gutierrez et al., 2023). Multiple signaling pathways can activate the PI3K-Akt signaling pathway, which can lead to increased transcription of NF-κB, and can further regulate the expression of cytokines, growth factors, and adhesion molecules (e.g., VEGF, IL6, VCAM-1), leading to cellular dysfunction (Li et al., 2023). The development of atherosclerosis is caused by impaired endothelial function, inflammation, and oxidative processes (Wolf and Ley, 2019). Fluid shear stress and atherosclerosis signaling pathway can activate PI3K-Akt signaling pathway, generate eNOS, and release NO by exerting stable laminar shear stress on vascular endothelial cells, thus participating in atherosclerosis, vasoconstriction, inflammatory response, cell proliferation, and angiogenesis, etc (Cheng et al., 2023). Lipid and atherosclerosis signaling pathways can be involved in the atherosclerotic process, allowing LDL-C to enter the vascular endothelium (Halasz et al., 2021). Vascular smooth muscle cells migrate and proliferate, which in turn form plaques. Damage to the vascular endothelium can cause an increase in blood pressure.

### 4.5 Cluster analysis and molecular docking: from prediction to validation

Cluster analysis was performed to analyze the common targets of UR-AP and HT, in order to find the key sub-networks and speculate the main targets. The results showed a high score for cluster 1 (score = 37.023). We predicted IL6, TNF, AKT1, VEGFA, IL1B, PTGS2, and PPARG as the main targets based on the degree. GO and KEGG pathway enrichment analysis of cluster 1 showed that cluster 1 may exert its efficacy by acting on the cell membrane and has an effect on lipopolysaccharides. Important signaling pathways including AGE-RAGE signaling pathway in diabetic complications, Fluid shear stress and atherosclerosis, TNF signaling pathway, IL-17 signaling pathway, Lipid and atherosclerosis were identified, which are mainly involved in atherosclerosis and inflammatory response.

Ultimately, we selected six core targets of UR-AP against HT and eight bioactive components for molecular docking to validate the interactions. The results showed strong binding interactions between the core targets and bioactive components with excellent stability.

### 4.6 Limitations and prospects for future research

This study presents an in-depth analysis of the mechanism of action of UR-AP in the context of hypertension, aiming to contribute significantly to TCM research, broaden clinical applications, and facilitate drug development. Despite its promise, challenges such as the intricate material basis of TCM and the gap between fundamental research and clinical application persist. Notably, the study faces limitations like the inability to perform experimental validations due to resource and financial constraints. Moving forward, our team is dedicated to keeping abreast of hypertension research advancements and plans to undertake animal or cellular experiments to further investigate UR-AP’s targets and mechanisms for hypertension. Additionally, pioneering efforts in network pharmacology will be made to elucidate the specific target modules of TCM’s effective population against hypertension, aiming to uncover TCM’s clinical efficacy and pharmacological mechanisms comprehensively.

## 5 Conclusion

This study systematically unraveled the intricate mechanisms underlying the therapeutic action of UR-AP in the management of HT, utilizing a novel integration of network pharmacology, cluster analysis, and molecular docking techniques. Key bioactive components from UR such as quercetin, kaempferol, and beta-sitosterol, along with Alisol B and alisol,b,23-acetate from AP, emerged as pivotal in mediating these beneficial effects. The study identified core targets including MAPK1, IL6, AKT1, VEGFA, EGFR, and TP53, highlighting their critical roles in the therapeutic efficacy of UR-AP.The investigation brought to light several vital signaling pathways, notably the AGE-RAGE signaling pathway, Fluid shear stress and atherosclerosis, Lipid and atherosclerosis, PI3K-Akt signaling pathway, and Calcium signaling pathway, which are instrumental in the action of UR-AP against HT. Our cluster analysis further pinpointed IL6, TNF, AKT1, and VEGFA as key targets, suggesting their crucial role in influencing cell membrane dynamics and responses to lipopolysaccharides. Predominantly, these pathways and targets are associated with the pathophysiology of atherosclerosis and inflammatory responses.Moreover, the molecular docking studies provided compelling evidence of strong binding interactions between the core targets and UR-AP’s major bioactive components, underscoring the potential of these compounds in HT therapy.

Building on this foundation, it is acknowledged that the study has its limitations, which have been discussed in a dedicated section within the document. Future research endeavors will seek to address these limitations through further experimental validations and explorations, particularly via animal models and cellular experiments, as well as employing cutting-edge techniques in network pharmacology to delve deeper into the action mechanisms of UR-AP and its potential applications in hypertension treatment.

## Data Availability

The original contributions presented in the study are included in the article/Supplementary Material, further inquiries can be directed to the corresponding authors.
